# Porcine Epidemic Diarrhea Virus Shedding and Antibody Response in Swine Farms: A Longitudinal Study

**DOI:** 10.3389/fmicb.2016.02009

**Published:** 2016-12-15

**Authors:** Cristina Bertasio, Enrico Giacomini, Massimiliano Lazzaro, Simona Perulli, Alice Papetti, Antonio Lavazza, Davide Lelli, Giovanni Alborali, Maria B. Boniotti

**Affiliations:** Istituto Zooprofilattico Sperimentale della Lombardia e Dell’Emilia Romagna (IZSLER)Brescia, Italy

**Keywords:** swine, porcine epidemic diarrhea virus, S INDEL PEDV, qRT-PCR, virus shedding

## Abstract

The porcine epidemic diarrhea virus (PEDV) causes an acute and highly contagious enteric disease characterized by severe enteritis, vomiting, watery diarrhea, and a high mortality rate in seronegative neonatal piglets. In the last few years, PED had a large economic impact on the swine industries in Asia and the US, and in 2014, the PEDV also re-emerged in Europe. Two main PEDV variants circulate worldwide but only the S INDEL variant, considered a mild strain, is spreading in Europe. To gain insights into the pathogenicity of this variant, its viral load and temporal shedding pattern were evaluated in piglets from infected farms. Quantitative real-time PCR (qPCR) targeting the *spike* gene, was validated according to the minimum information for quantitative real-time PCR experiments guidelines. The qPCR was applied to longitudinal studies conducted in four swine farms naturally infected with the PEDV S INDEL variant. Clinical data, fecal swabs, and blood samples were collected from 103 piglets at 15–30-day intervals for 2–5 months. On all four farms, diarrhea was observed in sows during gestation and in farrowing units, and the mortality rates of piglets were 18, 25, 30, and 35%. Different clinical pictures (0-50% of diarrhea positivity), viral titer levels (mean 5.3-7.2 log_10_ genome copies/mL), and antibody conditions (30-80% of positivity) were registered among sows on the four farms. The percentage of qPCR positive piglets varied greatly from the beginning (63–100%) to the end (0%) of the infection course. Clinical signs were present in 96% of the qPCR positive animals. Viral loads ranged from 8.5 log_10_ to 4 log_10_ genome copies/mL in suckling pigs at 3–6 days of age and were not statistically different among farms, despite the different patterns observed in sows. After 2–3 weeks, only a few piglets still showed detectable viral levels and clinical signs, and they developed antibody responses. Moreover, co-infections with other pathogens and biosecurity procedures limiting the circulation of the virus could have influenced the severity of PED infection. QPCR and clinical data were useful in understanding the dynamics of PEDV infections and, therefore, in implementing appropriate control measures.

## Introduction

Porcine epidemic diarrhea virus (PEDV) causes an acute and highly contagious enteric disease, which is characterized by severe enteritis, vomiting, watery diarrhea and a high mortality rate in neonatal piglets. Belonging to the family Coronaviridae, genus *Alphacoronavirus*, PEDV has a single-stranded, positive-sense RNA genome of ∼28 kb that encodes four structural proteins, spike (S), envelope, membrane, and nucleocapsid, and three nonstructural proteins (Kocherhans et al., 2001). In particular, the S protein is important in regulating interactions with specific cell receptor glycoproteins to mediate viral entry, inducing neutralizing antibodies (Bosch et al., 2003), growth adaptation in vitro and in virulence attenuation in vivo (Sato et al., 2011).

Since 2010, PED has caused large economic losses in the swine industries of Asia and North America (USA and Canada) (Stevenson et al., 2013; Kochhar, 2014). Based on the nucleotide sequence of the *S1* spike gene (Lee, 2015), two main genetic variants have been detected: a “highly virulent” strain, called “non-S INDEL (insertions and deletions)” (Vlasova et al., 2014; Sun et al., 2015), and a “mild” strain, called “S INDEL”, identified in pigs with mild clinical signs and no mortality (Vlasova et al., 2014; Wang et al., 2014).

Since 2014, in Europe, the non-S INDEL PEDV strain has been detected only in the Ukraine (Dastjerdi et al., 2015), while the S INDEL strain has spread throughout many countries including Germany, France, Belgium, Portugal and Italy (Grasland et al., 2015; Hanke et al., 2015; Mesquita et al., 2015; Theuns et al., 2015; Boniotti et al., 2016). All of the S INDEL PEDV European strains share 99% nucleotide identity with S INDEL PEDV OH851, but in contrast with field observation in the USA (Wang et al., 2014), outbreaks with high mortality rates in suckling piglets have been reported in Germany (Stadler et al., 2015) and Portugal (Mesquita et al., 2015).

In Italy, PED has been documented since 1997, with a few cases appearing per year. However, between 2005 and 2006, a severe PEDV epidemic occurred in Italy (Martelli et al., 2008), which was characterized by mortality rates in neonatal piglets of up to 34%, whereas it was very low in adults. During 2007–2012, only sporadic confirmed clinical cases of PED were reported (Boniotti et al., 2016). During this period, two swine coronavirus clades were identified. The first resembled the oldest global PEDV strain CV777 (PEDV/Italy/7239/2009) and the second resembled a new Transmissible Gastroenteritis Coronavirus/PEDV recombinant variant (SeCoV/Italy/213306/2009). During summer 2014, animals on two farms displaying mild clinical signs were detected as positive for PEDV by PCR (Boniotti et al., 2016), and at the beginning of 2015 a new severe epidemic wave occurred (Efsa Ahaw Panel, 2014).

Recently, the virulence of the S- INDEL strain has been evaluated and compared to the non-S INDEL strain through experimental infections (Lin et al., 2015; Yamamoto et al., 2015; Chen et al., 2016). In one study, 5-day-old piglets inoculated with an S INDEL PEDV strain (USA/IL/2014/20697) did not develop clinical signs and had only mild histopathological lesions (Chen et al., 2016). Lin et al. (2015) showed that the virulence of the S INDEL PEDV strain was lower than the non-S INDEL US PEDV based on a longer incubation time, a shorter duration of diarrhea, a lower percentage of infected enterocytes and a lower piglet mortality rate. However, the severity of clinical signs and mortality rates (0–75%) varied greatly among litters inoculated with the S INDEL strain. These variations were associated with piglet birth weights and the sow’s health and lactation status (Lin et al., 2015).

To evaluate differences in morbidity and the virulence of the S INDEL strains, field data are extremely important, as many concurrent factors, not reproducible in an experimental infection, can determine the infection severity. Presently, the limited available field observations (Wang et al., 2014; Mesquita et al., 2015; Stadler et al., 2015) indicated that the virulence level of S INDEL PEDV varied greatly. The different immunological states of infected piglets, due to lactogenic immunity, is a likely explanation of such variations.

Viral load is an important indicator in evaluating the virulence of a strain and the susceptibility of infected animals, and in understanding the mechanisms of viral transmission and circulation within the different farm units. At the moment, very limited data have been published on the PEDV viral load during an outbreak under field conditions (Bjustrom-Kraft et al., 2016).

In this study, we describe acute outbreaks of PED in three farrow-to-finish and one farrow-to-wean farms in Northern Italy. We conducted a longitudinal study by sampling the feces and blood of piglet groups from each farm at fixed intervals during a 2–5 months period, and then we determined PEDV shedding and the antibody presence. In particular, the quantification of PEDV in fecal samples was used to better describe the infection dynamics under field conditions.

## Materials and Methods

### Farms

Three farrow-to-finish and one farrow-to-wean farms located in the north of Italy were chosen for this study, which occurred from January to May 2015. Farms were selected based on the following criteria: location in the high density swine production area of the Po Valley, a short distance from the diagnostic laboratory to facilitate the conservation of samples and quick delivery, sudden onset of enteric clinical signs, mortality in newborn piglets clearly referable to PED and an absence of an anamnesis of PED on the farm.

In **Table 1**, data on the four farms are summarized. The PED clinical and epidemiological characteristics, as well as the disease course, for the four farms are recorded in Supplementary Table 1. At each sampling time, clinical evaluations of the farms were determined by vets from our Institute, recording the percentages, in four ranges, low (0–5%), medium (6–20%), high (21–50%), and very high (>50%), of diarrheic pigs among the different farm units (Supplementary Table 2). Fecal consistency was visually evaluated and scored using the following criteria: 0 = normal, 1 = formed and soft, 2 = semi-solid, 3 = watery, and 4 = presence of mucus and blood. The maximum observed fecal score attributed to each farm unit was recorded at each sampling time and reported in Supplementary Table 2.

**Table 1:** Farm production type, onset of outbreak, mortality rate, and animals enrolled in the longitudinal study.

Farm	Type	No. of sows	Onset of outbreak		% Mortality rate in suckling piglets	No. sampled animals	
			Date	Unit		Sows	Piglets
1	Farrow-to-finish	350	Jan-08	Fattening	25	10	19
2	Farrow-to-finish	350	Jan- 06	Fattening	30	10	24
3	Farrow-to-finish	650	Feb-15	Gestation	35	10	30
4	Farrow-to-wean	250	May-18	Delivery room	18	10	30

### Longitudinal Study

At 3–28 days from the first appearance of clinical signs on the four farms (F1-F4), 10 sows per farm, and 19, 24, 30, and 30 newborn piglets from F1, F2, F3, and F4, respectively, were selected from symptomatic litters, identified by ear tag and sampled every 2 weeks (samplings 1–3), 4-5 weeks (samplings 4-5) and after 7 weeks (sampling 6). The number of sampled animals decreased as the study continued due to mortality and animal sales (Supplementary Table 3). The sows were sampled only once at the beginning of the study. The presence of diarrheic piglets was recorded at each sampling time (Supplementary Table 3).

### PEDV RNA Extractions

Fecal and blood samples were collected from sows, whereas blood and rectal swabs were collected from piglets. Feces were diluted 1:10 (w/v) in minimum essential media (MEM). Rectal swabs were suspended in 1 mL of MEM, vortexed and incubated at 4°C for 30 min to allow the release of the feces from the cotton. Fecal suspensions were clarified by centrifugation for 10 min at 4,000 × g to eliminate fecal debris.

Viral RNA was extracted from 200 μL of sample using a commercial kit (NucleoMag^®^ Vet kit, Macherey-Nagel, Düren, Germany), according to the manufacturer’s instructions. An exogenous internal control RNA (IC) (Qiagen, Hilden, Germany), was added to specimens prior to RNA extraction to verify the success of the procedure and the absence of inhibitors. The extraction was carried out on the Biosprint 96 instrument (Qiagen) using the NucleoMag Vet 200 protocol. Nucleic acids were eluted into 100 μL of elution buffer and immediately subjected to RT-PCR or stored at -80°C until used.

### PEDV qRT-PCR Assay

Extracted viral RNA was subjected to a “one-step” RT-PCR assay using the commercial Quantifast Pathogen RT-PCR kit (Qiagen) with primers and probe targeting the S1 gene of PEDV that were previously developed at the University of Minnesota Veterinary Diagnostic Laboratory (2014), and are reported in **Table 2**. The optimum concentrations of primers and probe were deduced by titration experiments and are reported in **Table 2**. PCR reactions were performed on 5 μL of extracted viral RNA in a final volume of 25 μL, which also contained 5 μL of 5× PCR-master mix, 2.5 μL of a 10× internal control assay, 2.5 μL of each forward and reverse primer (final concentration 500 nM), 0.5 μL of PED_S probe (final concentration 200 nM), and 0.25 μL of enzyme mix. The PEDV RNA was reverse transcribed at 50°C for 20 min, followed by 1 cycle of Taq polymerase activation at 95°C for 5 min. Amplification consisted of 45 cycles at 95°C for 15 s and 60°C for 30 s. Amplification were performed on a CFX96 Touch Real-Time PCR Detection System (Bio-Rad Laboratories) and data were analyzed with the Software Bio-Rad CFX Manager 3.1, using the single threshold method for the Cq determination. An experiment was accepted when the Cq of the “no template control” (NTC) was >40 and the IC of a negative control was <35.

**Table 2:** Primer/probe sequences and concentrations (nM) used in quantitative real-time PCR (qPCR) assay.

	Sequence (5′-3′)	nt position^∗^	Conc
PED_S for	ACGTCCCTTTACTTTCAATTCACA	1846–1869	500
PED_S rev	TATACTTGGTACACACATCCAGAGTCA	1931–1957	500
PED_S Probe	FAM-TGAGTTGATTACTGGCACGCCTAAACCAC-BHQ1	1875–1903	200

*^∗^According to the published sequence of the porcine epidemic diarrhea virus (PEDV) *S1* gene, GenBank accession number DQ985739.1.*

#### Generation of the Standard Curve

We cloned a fragment of the S1 gene (nucleotide positions 1503–2153; GenBank DQ985739.1) into a pCR^®^ 2.1-TOPO^®^ vector (TOPO TA Cloning^®^ kit, Invitrogen) according to the manufacturer’s instructions. Plasmid DNA was linearized by restriction enzyme digestion and then subjected to transcription using the RiboMax Large Scale RNA Production System (Promega) to produce an RNA transcript.

The RNA concentration was determined using an Infinite^®^ 200 NanoQuant spectrophotometer (Tecan). A 10-fold serial dilution in nuclease-free water of ssRNA transcripts (2 × 10^6^ – 2 × 10^2^ copies/μL) was used to generate a standard curve and to quantify PEDV RNA in the samples. Triplicates of each dilution were run in each assay. The following equation:

(1) $$\begin{array}{c} {}^{x=10^{\frac{C_{q}-(y\;\;\mathrm{intercept})}{\mathrm{slope}}},} \end{array}$$

where x represents the genome copies/μL, was used to transform the samples’ Cq values into estimates of genome copies of PEDV RNA per mL of fecal homogenate.

#### qRT-PCR Performance Parameters

The qPCR assay used to quantify PEDV RNA was validated according to the minimum information for quantitative real-time PCR experiments guidelines (Bustin et al., 2009). Validation results are summarized in **Table 3**.

**Table 3:** Performance parameters of the PEDV qPCR assay.

Test	Result
Mean slope of four replicate standard curve	-3.3343
Calibration curve, *R^2^*	0.996
PCR Efficiency	99.5%
Linear dynamic range	2 × 10^0^-2 × 10^8^ genome copies/μL
Specificity	100%
LOD	2 genome copies/μL
LOQ	20 genome copies/μL
Cq variation at LOD	1.72%
Repeatability	±9.83% to ±12.75%
Reproducibility	12.82% (±7.85 to ±17.57)
Inhibition assay	3.15 cycles

*R^2^, linear correlation index; LOD, limit of detection; LOQ, limit of quantification.*

##### Specificity

To ascertain the specificity of the qPCR used in this study, we tested eight samples that were negative for PEDV and positive for different viral agents, including Transmissible Gastroenteritis Coronavirus, Rotavirus, Avian Influenza Virus H1N1, Reovirus, Encephalomyocarditis virus, Parvovirus, Suid Herpesvirus 1, and Porcine Reproductive and Respiratory Syndrome virus. The PCR assay amplified and detected only the intended virus (PEDV) and there was no evidence of viral cross-detection. Specificity is expressed as the percentage of negative reactions among the tested samples.

##### Limit of detection (LOD), limit of quantification (LOQ), and linearity

The limit of detection (LOD) and limit of quantification (LOQ) were determined by testing 10 replicates of a 10-fold serial dilution of ssRNA transcript, from an initial concentration of 2 × 10^5^ to a final concentration of 2 × 10^-2^ genome copies/μL (Supplementary Table 4). The LOD was calculated as the lowest concentration at which 100% of the positive samples are detected. The LOQ was defined as the lowest concentration of viral RNA that can be determined with acceptable precision (relative standard deviation ≤25%) under the stated conditions of the test.

The linear correlation index (*R*^2^) and the slope of the calibration curve were calculated using mean values from three replicates of four different runs. The reaction efficiency *(E)* under our experimental conditions was determined by the following formula:

$$\begin{array}{c} E=(10^{\frac{-1}{slope}}-1)\times 100\%. \end{array}$$

The dynamic range was determined by testing three replicates of a 10-fold serial dilution of ssRNA transcript, from an initial concentration of 2 × 10^8^ to a final concentration of 2 × 10^0^ genome copies/μL (Supplementary Figure 1).

##### Repeatability (Intra-assay Variance) and Reproducibility (Inter-assay Variance)

Repeatability was evaluated by analyzing three PEDV positive samples in triplicate in the same extraction and qPCR run (Supplementary Table 5). Intra-assay variance was expressed as the range of the relative standard deviation (RSD%) associated with copy number/μL. Reproducibility was determined by testing three PEDV-positive samples having different concentration levels. Samples were extracted and quantified on four different days (Supplementary Table 6). Inter-assay variance was expressed as mean RSD% with minimum and maximum values of copy numbers at each concentration level, calculated for the different concentration levels.

##### Inhibition assay

To verify the absence of contaminants that can cause the inhibition of qPCR assays after the RNA extraction procedure, the extracted RNAs of 10 specimens were diluted 1:10 (v/v) in water and subjected to qRT-PCR. Inhibition was evaluated by calculating the differences in mean Cq values between diluted and undiluted samples.

#### *S1 PEDV* Gene Sequencing

The *S1* gene sequences of PEDV positive samples on farm 1, were obtained from one sow and one piglet at the first sampling and from three piglets at 61 days of age, as described by Boniotti et al. (2016) (accession number: KY009940-KY009941).

### PEDV ELISA for Antibody Detection

An in-house PEDV whole virus ELISA was used. In brief, a PEDV strain CV777-based ELISA was developed and validated at IZSLER based on the previously described double-antibody sandwich ELISA protocol (Sozzi et al., 2010). The ELISA microplates were coated with the 1F12 capture monoclonal antibody (MAb). Serum samples diluted 1:2 or 1:4 were mixed with equal volumes of whole PEDV inactivated with ß-propiolactone and pre-incubated in an auxiliary microplate for 1 h at 37°C. Then, 50 μL of the pre-incubated mixtures were transferred into the 1F12 MAb-coated plate and the conjugated horseradish peroxidase MAb 4C3 was added. Following a further 1h incubation at 37°C, the plate was washed. The colorimetric reaction was performed, and optical densities (OD) were measured at 492 nm using an ELISA plate reader. Results were calculated by determining the absorbance value reduction, expressed as percentage of inhibition (PI) using the control wells as the reference. The antibody-blocking reaction was considered positive if the PI was ≥60%.

### Co-infections

For differential diagnoses and to investigate concomitant infections, fecal and serum samples were further investigated to detect Transmissible Gastroenteritis Coronavirus, Porcine Deltacoronavirus, swine Rotavirus A, B, C, and H, Porcine Reproductive and Respiratory Syndrome Virus; Influenza A Virus, *Escherichia coli, Clostridium perfringens*, and *Salmonella typhimurium*, as previously described (Kim et al., 2001; Marthaler et al., 2014a,b).

### Statistical Analyses

Statistical analyses were performed on quantitative data from fecal specimens of the first sampling both in sows and piglets, using the Kruskal-Wallis test and Dunn’s multiple comparisons test (GraphPad InStat Prism 6.0 software).

## Results

### Farm Clinical Assessments

At the beginning of the S INDEL PEDV epidemic wave in Northern Italy (January 2015), four farms with symptoms referable to PED were selected for a longitudinal study. Farm production type, onset of outbreak, mortality rate in suckling pigs and sampled animals for each farm, were described in **Table 1**. Severe clinical signs were observed during these outbreaks (Supplementary Table 1), with mortality rates in suckling piglets of 25, 30, 35, and 18% on F1, F2, F3, and F4, respectively (**Table 1**). PEDV quickly spread within F1, F2, and F3 to all of the units, but on F4 it only reached the gestation and delivery room (Supplementary Table 2). Watery diarrhea with presence of mucus and blood was observed in piglets of the four farms, but it was also present in gestation and farrowing sows. Growing and fattening animals from F1, F2, and F3 also showed severe clinical signs including watery diarrhea and anorexia. Vomiting was observed in fattening animals on F1. Dehydration was observed in litters on F1 and F3 and cachexia in litters on F1 and F4. Agalactia was present in the sows from F1, F2, and F3.

For differential diagnoses and to investigate concomitant infections, which potentially could impact the evolution of clinical signs and the course of the disease, fecal and serum samples were further investigated to detect other viral and bacterial pathogens. Rotavirus A was present in both the sows and piglets of F3, *E. coli*, expressing the virulence factor F4, in F2 and F3, *C. perfringens* in F1 and F3, and *S. typhimurium* in F3. However, no clinical signs, referable to a specific enteric disease, were present before the beginning of the longitudinal study.

### Longitudinal Study Outcomes

#### Symptoms

At the first sampling, watery diarrhea or soft diarrhea was observed in 30% (3/10), 0% (0/10), 50% (5/10), and 40% (4/10) of the sows from F1, F2, F3, and F4, respectively (**Table 4**). At the same time high percentages (68–100%) of 3–6-day-old piglets were observed to have diarrhea on all of the farms (**Table 5**). Clinical symptoms in piglets progressively disappeared at the following sampling times. However, a second outbreak of diarrhea was observed in 3 out of 16 animals (19%) at two months of age on F1.

**Table 4:** Diarrhea, PEDV detection in fecal samples, and cELISA results of sows.

	FARM 1			FARM 2			FARM 3			FARM 4		
Sow ID	Diarrhea	qRT-PCR	cELISA	Diarrhea	qRT-PCR	cELISA	Diarrhea	qRT-PCR	cELISA	Diarrhea	qRT-PCR	cELISA
1	+	+	+	-	+	+	+	+	-	-	+	-
2	-	+	-	-	+	+	+	+	-	+	+	-
3	-	+	+	-	+	+	-	+	-	-	+	+
4	+	+	+	-	+	+	+	+	-	+	-	-
5	-	+	+	-	NA	+	-	+	-	-	+	-
6	-	-	-	-	+	+	+	+	-	-	+	-
7	-	-	+	-	+	-	-	+	+	+	-	+
8	-	+	+	-	+	+	+	+	+	-	-	+
9	+	+	+	-	NA	-	-	+	-	+	+	-
10	-	+	+	-	+	-	-	+	+	-	+	NA

*+, presence/positive; -, absence/negative; NA, not available (missing sample).*

**Table 5:** Diarrhea, PEDV detection, and cELISA results for animals from 3 to 145 days of age.

Farm	Samplings (S)	No. of animals	Age (day)	Positivity (%)			No. animals^∗^	
				Diarrhea	qRT-PCR	cELISA	Died	NA
1	1	19	6	68.4	63.2	26.3	-	-
	2	18	23	N/A	N/A	88.9	1	-
	3	17	33	5.9	0	100	1	-
	4	16	61	18.8	18.8	100	-	1^a^
	5	12	97	0	8.3	100	-	5^b^
	6	8	145	0	0	100	-	4^b^
2	1	24	3	100	87.5	4.2	-	-
	2	24	18	29.2	16.7	91.7	-	-
	3	24	32	8.3	4.2	100	-	-
	4	20	67	0	5	90	3	1^a^
	5	18	103	0	0	82.4	-	2^b^
3	1	30	4	100	100	0	-	-
	2	30	17	10	10	83.3	-	-
	3	29	36	0	0	96.6	1	-
	4	15	72	0	0	93.3	-	14^b^
4	1	30	3	90	86.7	20	-	-
	2	22	14	36.4	22.7	95.5	5	3^a^
	3	22	30	0	0	86.4	-	-
	4	21	63	0	0	81	4	6^a^

*Animals died or were not available (NA). The number of sampled animals decreased as the study continued due to mortality, earmarks lost (NA^a^), and animal sales (NA^b^).

#### PEDV RNA Detection in Sows

**FIGURE 1:**
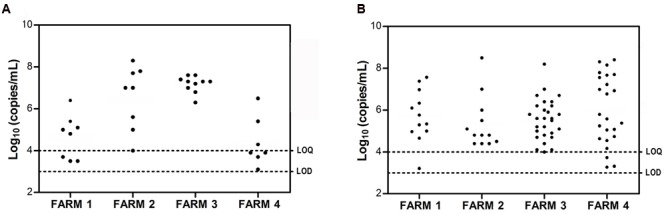
**Fecal porcine epidemic diarrhea virus (PEDV) RNA shedding of sows (A)** and of 3–6-day-old piglets **(B)** in each farm as determined by qRT-PCR. Individual viral titers are expressed as log_10_ genome copies/mL. Limit of detection (LOD) and limit of quantification (LOQ) values are indicated.

Samples were only taken from sows at the initial sampling time, which occurred at 7, 28, 3, and 19 days after the onset of symptoms on F1, F2, F3, and F4, respectively. PEDV RNA was detected in 8/10 sows on F1, 8/8 sows on F2, 10/10 on F3 and 7/10 on F4 (**Table 4**). The highest fecal PEDV RNA shedding titers were detected on F2 and F3 (8.3 and 7.6 log_10_ genome copies per mL of fecal homogenate), with a mean titer among shedding animals of 6.5 and 7.2 log_10_ copies/mL, respectively (**Figure 1A**). Mean lower titers were observed in F1 and F4 animals (5.3 and 5.4 log_10_ copies/mL, respectively) where the highest titers were 6.4 and 6.5 log_10_ copies/mL, respectively.

#### PEDV RNA Detection in Piglets

The fecal viral shedding in piglets from the four farms is summarized in **Table 5** and **Figure 1B**. All of the farms had a similar virus-shedding pattern, with high percentages of PEDV PCR positive 3–6-day-old animals (Sampling 1) that decreased in 14–18-day-old animals (Sampling 2) and was no longer present in 30–36-day-old animals (Sampling 3) to the end of the study period (**Table 5**). Furthermore, on F1, a second peak of viral shedding was detected in weaning piglets at 61 days of age (Sampling 4). Two weaning piglets did not show detectable viral shedding in the previous samplings. Interestingly, the presence of different strains was assessed by sequencing the *S1* gene of positive samples collected at the first and forth samplings. Two genetic variants with a single amino acid substitution (156E > G) were detected at Samplings 1 and 4. On F2, 87.5% of 3-day-old piglets were PCR positive, four of them (16.7%) were positive at 18 days of age (Sampling 2) and only one animal was still positive at 32-days old (Sampling 3). Moreover, one pig showed intermittent PEDV shedding, being PCR positive at the first sampling (3 days after birth), negative at 18- and 32-days old (Samplings 2 and 3, respectively) and again positive at 67-days old (Sampling 4) (Supplementary Table 3). On F3, all of the 4-day-old piglets were PCR positive using rectal swab samples but only three of them remained positive at 17-days old (Sampling 2). At the following sampling times, when they were 36- and 72-days old, they were all PCR negative. On F4, 86.7% of 3-day-old piglets were PCR positive but viral shedding was not detected in 30-day-old piglets through the end of the study. The highest fecal PEDV RNA shedding titer was observed in 3–6 day-old piglets with mean values (among shedding animals) of 5.9, 5.6, 5.6, and 6.2 log_10_ copies/mL on F1, F2, F3, and F4, respectively (**Figure 1B**; Supplementary Table 3). No significant differences were statistically evident among the farms. The titer values observed in 14–18-day-old piglets were 5.9 (one animal), 4.8 (mean value of two animals), 4.9 (mean value of three animals) log_10_ copies/mL on F2, F3, and F4, respectively (Supplementary Table 3).

#### PEDV Antibody Detection in Sows and Piglets

PEDV-antibodies were detected in 8/10, 7/10, 3/10, and 3/9 sows on F1, F2, F3, and F4, respectively (**Table 4**). In newborn piglets, PEDV-antibodies were detected in 26, 4, 0, and 20% of animals from F1, F2, F3, and F4, respectively (**Table 5**). In total, 54% of the sows had anti-PEDV antibodies at delivery but only a few piglets (3%) showed detectable antibodies and a lack of clinical signs at 3–6 days of age. Most of the piglets on the four farms developed antibody responses within 3 weeks of age, and they remained stable until the end of the study (60–100 days of age).

## Discussion

In the last few years, PED had a large economic impact on the swine industries in Asia and the US, and in 2014, the PEDV also re-emerged in Europe. Two main PEDV variants circulate worldwide but only the S INDEL variant, considered a mild strain, is spreading in Europe. During summer 2014, animals with mild clinical signs on two farms were detected as PCR positive for PEDV in Northern Italy (Boniotti et al., 2016). Thereafter, a new severe PED epidemic wave occurred. To gain insights into the pathogenicity of this variant, we described the results of a longitudinal study conducted during acute outbreaks of PED in three farrow-to-finish (F1-F3) and one farrow-to-wean farm (F4), occurred in the beginning of 2015.

On the four farms, the mortality rates of suckling piglets were high (25, 30, 35 and 18% on F1, F2, F3 and F4, respectively) (**Table 1**). However, they did not reach the percentages observed in the US, caused by the original PEDV strain (>50%) (Alvarez et al., 2015). Moreover, the four farms showed high percentages (21-50, >50%) of diarrheic animals, in particular, in all the units of the F1, in piglets of F2 and in sows and piglets of F3 and F4, but differences among farms were also observed. The course of PED was particularly severe on F1, where we observed a high percentage of animals with diarrhea in suckling, weaning and fattening animals (Supplementary Table 2). Moreover, the virus appeared to circulate longer on this farm since positive animals were detected even 60 and 100 days after the first PEDV detection within the farm. In particular, at 60 days of age a second peak of viral shedding was observed in three animals. The *S1* gene sequences of the strain identified in these animals showed a different genetic variant compared with the strain circulating at the onset of the clinical signs. Thus, an additional introduction of a second viral strain likely occurred. Such a situation, as well as the quick spread of PEDV on one farm to the different units, could be the direct consequence of the application of poor biosecurity measures, incorrect management and/or the limited efficacy of disinfection procedures.

The different patterns and severity of clinical signs observed on the four farms may have resulted from the concurrent effects of co-infections with other pathogens. In particular, Rotavirus, with a well-known pathogenic aptitude, which is mainly dose-dependent, could have had a synergistic effect with PEDV on F3, causing a slight change in the course of the disease in sows and litters after the first week, and predisposing animals to secondary infections, such as those caused by *E. coli F4, C. perfrigens*, and *S. typhimurium*.

In this study, we determined the fecal PEDV shedding in sows and in their piglets from 3 to 6 days to 60-100 days of age. In particular, we selected groups of 10 sows and 19–30 piglets on each farm. None of the farms had reported PEDV infections prior to this study, and thus, we can surmise that the sows had been recently infected at the time of their enrollment in the study, while piglets were infected after birth, through contact with infected sows. More than 80% of the sows showed detectable levels of PEDV at the first sampling but only the 30% showed diarrhea. The severity of clinical signs caused by PEDV is age-dependent (Jung et al., 2016). In adult animals, clinical signs are usually milder or completely absent. Moreover, in our study, PEDV exposure dose and the gestational stage of the sows could have influenced their health status at the moment of delivery in terms of clinical signs, viral loads and PEDV antibody level. On F1 and F2, where the onset of PEDV symptoms occurred in the fattening unit, 8 and 7 out of 10 sows, respectively, had PEDV antibodies. On F3, where the onset of PEDV symptoms occurred during the gestation period, 5 out of 10 sows had diarrhea but only 3 had already developed PEDV antibodies. On this farm, all of the sows showed high mean viral loads (7.2 log_10_ copies/mL) as determined by PEDV RNA. Sows on F3 probably had less time to develop antibodies and transmit them to their litters through the colostrum. In fact, all of the piglets were infected and showed high titers of PEDV RNA in fecal materials. Similarly, on F4, where the onset of the outbreak took place in the delivery room, 3 out of 9 sows showed detectable levels of PEDV antibodies.

At the first sampling, a high percentage (63–100%) of the 3–6-day-old piglets from all of the farms were positive by qPCR. Despite the different immunity levels among the sows on the four farms, and the different proportions of infected piglets, the quantitative PEDV RNA results in piglets were not statistically different among farms. As proposed by other authors, we assumed that once the pigs were infected and viral replication began, the initial viral dose appeared to have little impact, at the group level, on the average amount of fecal shedding (Thomas et al., 2015).

At 2 weeks of age, the proportion of PCR positive piglets decreased to 10–22.7%, and at 1 month of age only one animal was positive. The PEDV RNA titer also decreased with time, confirming that PED is characterized by high PEDV fecal shedding titers a few days post infection and the titers tend to decrease after 1 week. Intermittent shedding can be observed until 60 days post infection, as shown in F1 and F2, and such variations in excretion levels and viral loads could be determined by poor management and a lack of biosecurity measures, but it could also be due to a new introduction of the virus, as likely occurred on F1.

For immunity responses, 100% of the animals on F1 showed detectable levels of PEDV antibodies up to the end of the study (145 days of age). On F2, F3, and F4, the percentage of seropositive animals decreased slightly starting from 80 days of age. The longer and biphasic circulation of the virus on F1 might have favored subsequent exposures of the animals to the virus, and thus, a more durable immunity. The likely outcome of a massive outbreak of PEDV within one herd is a diffuse and long-lasting immunity. Indeed, especially in farrow-to-finish farms, such herd immunity and, particularly, the maintenance of seropositive sows could be an important tool to prevent severe cases of PED in newborn piglets by transferring passive maternal immunity with colostrum. Goede et al. (2015) reported that durable lactogenic immunity was present in sows previously exposed (7 months) to a S INDEL strain of PEDV and that this immunity induced cross-protection to an original virulent PEDV. Cumbersomely, in this study we did not plan to investigate the presence of maternal antibodies (IgG and IgA) in the colostrum or to register the piglets’ birth weight, two important factors influencing the protective maternal immunity. However, considering the high percentage of diarrheic and PEDV positive piglets, and the presence of clinical signs in animals of different ages, including sows, we can suppose that these farms were originally naïve with regard to PEDV infection, and thus the lactogenic antibodies were not present at all, or anyway sufficient to effectively protect piglets from infection. Thus, future management of PEDV infection in farrow-to-finish or farrow-to-wean farms cannot disregard to periodically check and evaluate the IgA and IgG titers in sow sera and in the colostrum.

## Conclusion

Determining the viral loads and shedding rates of PEDV in real field situations during outbreaks is important in evaluating the virulence of a strain and in predicting the susceptibility of infected animals, at different ages and in the various farm units, within a herd. At the moment, very limited data have been published on the PEDV viral load during an outbreak under field conditions (Bjustrom-Kraft et al., 2016). The qPCR assay used in this study was validated according to the minimum information for quantitative real-time PCR experiments guidelines (Bustin et al., 2009; **Table 3**; Supplementary Table 7). The method validation is an important requirement to allow the comparison of quantitative results from different studies, which would be otherwise difficult to achieve.

Moreover, understanding the mechanisms of viral transmission and circulation within the farm can be useful in implementing appropriate control measures on the farm to limit the infections spread. Considering the ability of PEDV to be dispersed rapidly through fecal contamination, particular attention should be paid to biosecurity and hygiene measures, such as the proper disinfection of equipment and sites, manure disposal, suitable procedures for the movements of animals within the farm, and the use of disposable clothes and shoes for personnel, staff and visitors.

Longitudinal field studies examining natural infections are comparatively uncommon amongst reports of PEDV in comparison to the several experimental studies already performed. In fact, many unforeseen events can adversely affect the success of this kind of study: dead of animals, lost of the earmark, selling or moving of the animals due to unexpected need of the farmer. These practical hitches could negatively influence the sampling procedures (e.g., respect of time points) and data registration. Therefore, by planning these “observational” studies, so tight inclusion criteria cannot be established and limited field data could be successfully registered. On the other hand, the longitudinal studies, directly conducted in natural outbreaks, are inclusive of the several “farm factors and field effects” which are very difficult to reproduce in experimental trials, and thus, the obtained results, being more reliable and adherent to real conditions could be effectively used in risk analysis and for defining control strategies. In conclusion, longitudinal studies conducted under field conditions during and after a PED outbreak could be useful in determining the level of immunity acquired at the herd level and may be integrated with the data acquired from experimental infections. They provide an added value, in the possibility to study and evaluate the effects of co-factors, such as other infectious agents, and management and environmental conditions, on the evolution and epidemiology of the disease.

## Ethics Statement

The study was exempt of ethical approval procedures because animal samplings were performed during the routinely diagnostic procedures in naturally infected farms.

## Author Contributions

Performed experiments: CB, EG, ML, SP. Analyzed data: CB. Conceived and designed experiments: All authors. Wrote and revised the paper: MB, CB, AL, GA. All authors have approved the final version of the article.

## Conflict of Interest Statement

The authors declare that the research was conducted in the absence of any commercial or financial relationships that could be construed as a potential conflict of interest.
