# Supplementary material for: Porcine Epidemic Diarrhea Virus Shedding and Antibody Response in Swine Farms: A Longitudinal Study
Source: Front Microbiol. 2016 Dec 15;7:2009. doi: 10.3389/fmicb.2016.02009 (PMC5156881; doi:10.3389/fmicb.2016.02009)
Supplement: Supplementary file 1 [file Data_Sheet_1.pdf]

Supplementary Material

Porcine Epidemic Diarrhea Virus Shedding and Antibody Response in Swine Farms: a Longitudinal Study

Cristina Bertasio [1], Enrico Giacomini [1], Massimiliano Lazzaro [1], Simona Perulli[1], Alice Papetti[1], Antonio Lavazza [1], Davide Lelli [1], Giovanni Alborali [1], Maria Beatrice Boniotti [1]*

[1]Istituto Zooprofilattico Sperimentale della Lombardia ed Emilia Romagna, Brescia, Italy

*** Correspondence:** Maria Beatrice Boniotti, Istituto Zooprofilattico Sperimentale della Lombardia ed Emilia Romagna, Brescia, Italy

mariabeatrice.boniotti@izsler.it

Supplementary Figures

Supplementary Figure 1. Dynamic range of the standard curve for the quantitative PEDV *S1* gene-based qRT-PCR assay. The Cq values of the10-fold stock PEDV ssRNA transcript ($2 \times 10^8 – 2 \times 10^0$ copies/µL) solutions are dependent on the log values of the transcript amounts.

Supplementary Tables

Supplementary Table 1. Order of appearance of PED symptoms in the different units of the farm.

Farm	DPI	Unit	Symptoms					
			diarrhea	vomiting	anorexia	dehydration	cachexia	agalactia
1	0	fattening A	x	x	x			
	1-2	gestation	x					
	1-2	delivery (litters)	x			x	x	
	1-2	delivery (sows)	x					x
	3	weaning	x					
	3	growing	x					
	5	fattening B	x					
2	0	fattening	x		x			
	3	gestation	x					
	3	delivery (litters)	x		x			
	3	delivery (sows)	x		x			x
	3	weaning	x					
	4-5	growing	x					
3	0	gestation	x		x			
	1	delivery (litters)	x		x	x		
	1	delivery (sows)	x					x
	5	weaning	x					
	5	growing	x					
	5	fattening	x					
4	0	delivery (litters)	x				x	
	0	delivery (sows)	x					
	3	gestation	x					

DPI (Days Post Infection): days from the first appearance of the infection in the farm, x: presence of symptoms.

Supplementary Table 2. Maximum fecal score and percentage of diarrheic animals per farm.

			Gestation unit		Delivery room				Weaning unit		Growing unit		Fattening unit A		Fattening unit B	
					Sows		Piglets									
Farm	Sampling time	Date	Fecal score	Diarrheic animals (%)	Fecal score	Diarrheic animals (%)	Fecal score	Diarrheic animals (%)	Fecal score	Diarrheic animals (%)	Fecal score	Diarrheic animals (%)	Fecal score	Diarrheic animals (%)	Fecal score	Diarrheic animals (%)
	preliminary	13 Jan	4	21-50	2	6-20	4	>50	2	0-5	4	21-50	4	>50	0	0
	1	16 Jan	3	21-50	2	5	4	>50*	3	21-50	4	21-50	4	>50	0	0
	2	2 Feb	0	0	0	0	4	6-20*	0	0	0	0	2	0-5	2	6-20
1	3	12 Feb	0	0	0	0	4	21-50	0	0*	0	0	0	0	2	21-50
	4	12 Mar	0	0	0	0	0	0	2	0-5*	2	0-5	2	0-5	2	0-5
	5	17 Apr	0	0	0	0	0	0	0	0	0	0*	0	0	0	-
	6	4 June	0	0	0	0	0	0	0	0	0	0	0	0*	0	-*
	preliminary	31 Jan	4	6-20	2	0-5	4	>50	4	6-20	4	6-20	4	6-20	/	/
	1	3 Feb	3	0-5	0	0	4	21-50*	3	0-5	3	0-5	4	6-20	/	/
2	2	18 Feb	0	0	2	0-5	2	0-5*	0	0	0	0	0	0	/	/
	3	4 Mar	0	0	0	0	0	0	0	0*	0	0	0	0	/	/
	4	8 Apr	0	0	0	0	0	0	0	0*	0	0	0	0	/	/
	5	14 May	0	0	0	0	0	0	0	0	0	0*	0	0	/	/
	preliminary	18 Feb	4	0-5	4	0-5	4	6-20	0	0	0	0	0	0	/	/
	1	20 Feb	4	6-20	4	21-50	4	>50*	2	0-5	4	0-5	4	0-5	/	/
3	2	5 Mar	0	0	0	0	0	0*	0	0	0	0	0	0	/	/
	3	24 Mar	0	0	0	0	0	0	0	0	0	0	0	0	/	/
	4	29 Apr	0	0	0	0	0	0	0	0	0	0	0	0	/	/
	preliminary	18 May	2	0-5	2	0-5	3	6-20	0	0	/	/	/	/	/	/
	1	6 June	0	0	4	21-50	4	21-50*	0	0	/	/	/	/	/	/
4	2	17June	0	0	2	0-5	0	0*	0	0	/	/	/	/	/	/
	3	23 July	0	0	0	0	0	0	0	0*	/	/	/	/	/	/
	4	5 Aug	0	0	0	0	0	0	0	0*	/	/	/	/	/	/

*indicates the location of animals involved in the study; “/” indicates that samples were not available

Supplementary Table 3. Symptoms observation, viral titers in fecal homogenates and cELISA results for animals involved in the longitudinal study.

Farm	Animal ID	1° SAMPLING			2° SAMPLING			3° SAMPLING			4° SAMPLING			5° SAMPLING			6° SAMPLING		
		**Diarrhea**	**PEDv copies/mL**	**cELISA**	**Diarrhea**	**PEDv copies/mL**	**cELISA**	**Diarrhea**	**PEDv copies/mL**	**cELISA**	**Diarrhea**	**PEDv copies/mL**	**cELISA**	**Diarrhea**	**PEDv copies/mL**	**cELISA**	**Diarrhea**	**PEDv copies/mL**	**cELISA**
1	1	+	< LOQ	+	NA	NA	+	+	-	+	-	-	+	-	-	+	-	-	+
1	2	-	-	-	NA	NA	+	-	-	+	-	-	+	-	-	+	-	-	+
1	3	+	-	-	NA	NA	+	-	-	+	-	-	+	-	-	+	-	-	+
1	4	-	2.00E+05	-	NA	NA	+	-	-	+	-	-	+	-	-	+	-	-	+
1	5	-	-	-	NA	NA	+	-	-	+	+	< LOD	+	-	-	+	-	-	+
1	6	+	1.00E+05	-	NA	NA	+	-	-	+	-	-	+	-	-	+	-	-	+
1	7	+	-	+	NA	NA	+	-	-	+	-	-	+	S	S	S	S	S	S
1	8	+	5.90E+05	-	NA	NA	+	-	-	+	-	-	+	-	-	+	S	S	S
1	9	+	1.20E+06	-	NA	NA	+	-	-	+	-	-	NA	-	-	+	S	S	S
1	10	+	9.50E+06	+	NA	NA	+	-	-	+	-	-	+	-	-	+	S	S	S
1	11	+	2.20E+05	-	NA	NA	+	D	D	D	D	D	D	D	D	D	D	D	D
1	12	+	4.60E+04	+	NA	NA	+	-	-	+	-	-	+	-	-	+	-	-	+
1	13	+	2.40E+07	+	NA	NA	+	-	-	+	NA	NA	NA	-	-	+	-	-	+
1	14	+	9.50E+04	-	NA	NA	+	-	-	+	-	4.90E+04	+	S	S	S	S	S	S
1	15	-	-	-	NA	NA	+	-	-	+	+	9.40E+05	+	S	S	S	S	S	S
1	16	+	-	-	NA	NA	-	-	-	+	+	7.90E+04	+	S	S	S	S	S	S
1	17	-	3.70E+07	-	NA	NA	+	-	-	+	-	-	+	-	< LOQ	+	S	S	S
1	18	+	2.10E+06	-	NA	NA	-	-	-	+	-	-	+	S	S	S	S	S	S
1	19	-	-	-	NA	NA	D	D	D	D	D	D	D	D	D	D	D	D	D
2	1	+	-	-	+	< LOQ	+	-	-	+	-	-	-	-	-	NA	/	/	/
2	2	+	3.30E+05	-	-	-	+	-	-	+	-	-	+	-	-	+	/	/	/
2	3	+	1.10E+06	-	-	-	+	-	-	+	-	-	+	-	-	+	/	/	/
2	4	+	1.10E+07	-	-	-	+	-	-	+	-	-	+	-	-	+	/	/	/
2	5	+	3.20E+04	-	-	-	+	-	-	+	-	-	+	-	-	+	/	/	/
2	6	+	3.30E+08	-	+	-	+	-	-	+	-	3.00E+04	+	-	-	+	/	/	/
2	7	+	1.20E+05	-	+	< LOQ	-	-	-	+	-	-	+	S	S	S	/	/	/
2	8	+	-	-	+	8.30E+05	-	-	-	+	D	D	D	D	D	D	/	/	/
2	9	+	7.00E+04	-	-	-	+	-	-	+	-	-	+	-	-	-	/	/	/
2	10	+	2.60E+04	-	-	-	+	-	-	+	D	D	D	D	D	D	/	/	/
2	11	+	7.00E+04	-	-	-	+	-	-	+	-	-	+	-	-	+	/	/	/
2	12	+	2.60E+04	-	-	-	+	-	-	+	-	-	+	-	-	+	/	/	/
2	13	+	7.00E+04	-	-	-	+	-	-	+	D	D	D	D	D	D	/	/	/
2	14	+	2.60E+04	-	+	-	+	-	-	+	-	-	+	-	-	+	/	/	/
2	15	+	-	-	-	-	+	-	-	+	-	-	+	-	-	+	/	/	/
2	16	+	7.30E+04	-	-	-	+	-	-	+	D	D	D	D	D	-	/	/	/

2	17	+	1.00E+04	-	-	-	+	-	-	+	-	-	+	-	-	+	/	/	/
2	18	+	1.40E+05	-	+	-	+	+	-	+	-	-	+	-	-	+	/	/	/
2	19	+	1.00E+08	-	-	-	+	-	-	+	-	-	+	-	-	+	/	/	/
2	20	+	4.50E+04	-	-	-	+	-	-	+	-	-	+	-	-	+	/	/	/
2	21	+	3.60E+05	-	-	-	+	-	-	+	-	-	+	S	S	S	/	/	/
2	22	+	< LOQ	-	-	-	+	-	-	+	-	-	+	-	-	-	/	/	/
2	23	+	1.30E+08	-	-	-	+	-	-	+	-	-	+	-	-	+	/	/	/
2	24	+	6.40E+06	-	+	< LOQ	+	+	1.10E+04	+	-	-	-	-	-	NA	/	/	/
3	1	+	7.80E+04	-	-	-	+	-	-	+	-	-	-	/	/	/	/	/	/
3	2	+	1.50E+06	-	-	-	+	D	D	D	D	D	D	/	/	/	/	/	/
3	3	+	3.50E+05	-	-	-	+	-	-	+	-	-	+	/	/	/	/	/	/
3	4	+	5.60E+06	-	-	-	+	-	-	+	S	S	S	/	/	/	/	/	/
3	5	+	6.80E+05	-	-	-	+	-	-	+	S	S	S	/	/	/	/	/	/
3	6	+	1.20E+04	-	-	-	+	-	-	+	-	-	+	/	/	/	/	/	/
3	7	+	9.10E+04	-	-	-	+	-	-	+	S	S	S	/	/	/	/	/	/
3	8	+	1.40E+04	-	-	-	+	-	-	+	-	-	+	/	/	/	/	/	/
3	9	+	< LOQ	-	-	-	+	-	-	+	S	S	S	/	/	/	/	/	/
3	10	+	5.10E+04	-	-	-	-	-	-	+	S	S	S	/	/	/	/	/	/
3	11	+	4.20E+05	-	-	-	+	-	-	+	S	S	S	/	/	/	/	/	/
3	12	+	5.40E+04	-	-	-	+	-	-	-	S	S	S	/	/	/	/	/	/
3	13	+	< LOQ	-	-	-	+	-	-	+	S	S	S	/	/	/	/	/	/
3	14	+	5.50E+06	-	-	-	+	-	-	+	-	-	+	/	/	/	/	/	/
3	15	+	2.70E+06	-	-	-	+	-	-	+	S	S	S	/	/	/	/	/	/
3	16	+	2.40E+06	-	-	-	-	-	-	+	S	S	S	/	/	/	/	/	/
3	17	+	1.20E+05	-	+	3..00E+05	+	-	-	+	-	-	+	/	/	/	/	/	/
3	18	+	1.50E+05	-	-	-	-	-	-	+	S	S	S	/	/	/	/	/	/
3	19	+	6.90E+05	-	-	-	-	-	-	+	-	-	+	/	/	/	/	/	/
3	20	+	2.50E+04	-	-	-	+	-	-	+	S	S	S	/	/	/	/	/	/
3	21	+	4.10E+05	-	-	-	+	-	-	+	-	-	+	/	/	/	/	/	/
3	22	+	6.40E+04	-	-	-	+	-	-	+	-	-	+	/	/	/	/	/	/
3	23	+	1.70E+05	-	-	-	-	-	-	+	-	-	+	/	/	/	/	/	/
3	24	+	1.10E+04	-	-	-	+	-	-	+	S	S	S	/	/	/	/	/	/
3	25	+	1.40E+06	-	+	1.10E+04	+	-	-	+	-	-	+	/	/	/	/	/	/
3	26	+	9.00E+06	-	+	6.70E+03	+	-	-	+	S	S	S	/	/	/	/	/	/
3	27	+	8.00E+05	-	-	-	+	-	-	+	-	-	+	/	/	/	/	/	/
3	28	+	1.50E+08	-	-	-	+	-	-	+	-	-	+	/	/	/	/	/	/
3	29	+	9.60E+05	-	-	-	+	-	-	+	-	-	+	/	/	/	/	/	/
3	30	+	4.00E+05	-	-	-	+	-	-	+	-	-	+	/	/	/	/	/	/
4	1	+	1.70E+07	-	-	-	+	-	-	+	-	-	+	/	/	/	/	/	/
4	2	+	5.90E+06	-	D	D	D	D	D	D	D	D	D	/	/	/	/	/	/
4	3	+	8.60E+06	-	D	D	D	D	D	D	D	D	D	/	/	/	/	/	/
4	4	+	1.10E+05	-	D	D	D	D	D	D	D	D	D	/	/	/	/	/	/

4	5	+	6.20E+07	-	+	-	+	-	-	+	-	-	+	/	/	/	/	/	/
4	6	+	1.50E+04	-	+	1.80E+04	+	-	-	-	-	-	+	/	/	/	/	/	/
4	7	+	3.60E+07	-	-	-	+	-	-	+	-	-	+	/	/	/	/	/	/
4	8	+	2.40E+05	-	-	-	+	-	-	+	-	-	+	/	/	/	/	/	/
4	9	+	< LOQ	-	-	-	+	-	-	+	-	-	+	/	/	/	/	/	/
4	10	+	1.80E+05	-	D	D	D	D	D	D	D	D	D	/	/	/	/	/	/
4	11	+	9.80E+06	-	-	-	+	-	-	+	D	D	D	/	/	/	/	/	/
4	12	+	6.40E+05	-	D	D	D	D	D	D	D	D	D	/	/	/	/	/	/
4	13	+	1.40E+08	-	NA	NA	NA	NA	NA	NA	-	-	+	/	/	/	/	/	/
4	14	+	< LOQ	-	NA	NA	NA	NA	NA	NA	-	-	+	/	/	/	/	/	/
4	15	+	4.70E+07	-	NA	NA	NA	NA	NA	NA	-	-	+	/	/	/	/	/	/
4	16	+	1.20E+05	-	-	-	+	-	-	+	-	-	+	/	/	/	/	/	/
4	17	+	5.60E+04	-	-	-	+	-	-	+	-	-	+	/	/	/	/	/	/
4	18	+	5.00E+07	-	-	-	+	-	-	+	-	-	+	/	/	/	/	/	/
4	19	+	< LOQ	+	-	-	+	-	-	+	-	-	-	/	/	/	/	/	/
4	20	+	-	-	-	-	-	-	-	-	-	-	-	/	/	/	/	/	/
4	21	-	-	+	-	-	+	-	-	-	-	-	-	/	/	/	/	/	/
4	22	-	-	+	-	-	+	-	-	+	-	-	+	/	/	/	/	/	/
4	23	+	4.20E+04	+	-	-	+	-	-	+	D	D	D	/	/	/	/	/	/
4	24	-	-	+	-	-	+	-	-	+	D	D	D	/	/	/	/	/	/
4	25	+	< LOQ	-	+	1.80E+06	+	-	-	+	D	D	D	/	/	/	/	/	/
4	26	+	< LOQ	-	+	-	+	-	-	+	-	-	+	/	/	/	/	/	/
4	27	+	3.80E+05	-	+	< LOQ	+	-	-	+	-	-	-	/	/	/	/	/	/
4	28	+	2.10E+08	-	+	< LOQ	+	-	-	+	-	-	+	/	/	/	/	/	/
4	29	+	2.60E+08	-	+	< LOD	+	-	-	+	-	-	+	/	/	/	/	/	/
4	30	+	3.60E+04	+	+	1.90E+04	+	-	-	+	-	-	+	/	/	/	/	/	/

+: presence/positive; -: absence/negative; NA: not available; D: died; S: sold.

Supplementary Table 4. Determination of the LOD and the LOQ.

Replicate	Theoretical ssRNA transcript copies/µL															
	$2x10^5$		$2x10^4$		$2x10^3$		$2x10^2$		$2x10^1$		$2x10^0$		$2x10^{-1}$		$2x10^{-2}$	
	**Cq**	**copies/µL**	**Cq**	**copies/µL**	**Cq**	**copies/µL**	**Cq**	**copies/µL**	**Cq**	**copies/µL**	**Cq**	**copies/µL**	**Cq**	**copies/µL**	**Cq**	**copies/µL**
1	19.05	2.46E+05	23.09	1.56E+04	25.92	2.27E+03	29.78	1.63E+02	33.02	1.79E+01	37.05	1.14E+00	38.12	5.50E-01	ND	0.00E+00
2	19.04	2.48E+05	23.1	1.55E+04	26.09	2.02E+03	29.44	2.05E+02	33.32	1.46E+01	35.88	2.54E+00	39.76	1.80E-01	37.45	5.75E-01
3	19.51	1.80E+05	23.28	1.37E+04	25.76	2.53E+03	29.34	2.20E+02	33.76	1.08E+01	35.45	3.40E+00	37.78	6.94E-01	ND	0.00E+00
4	19.06	2.45E+05	23.18	1.47E+04	25.79	2.48E+03	29.51	1.96E+02	33.02	1.79E+01	35.65	2.97E+00	ND	0.00E+00	ND	0.00E+00
5	18.68	3.17E+05	23.27	1.38E+04	25.89	2.32E+03	29.54	1.92E+02	33.49	1.30E+01	35.98	2.37E+00	ND	0.00E+00	ND	0.00E+00
6	18.91	2.71E+05	23.28	1.37E+04	25.9	2.30E+03	29.22	2.39E+02	33.26	1.52E+01	37.05	1.14E+00	37.52	8.29E-01	ND	0.00E+00
7	19.02	2.51E+05	24.01	8.35E+03	25.65	2.73E+03	29.51	1.96E+02	32.59	2.40E+01	35.99	2.35E+00	37.54	8.18E-01	ND	0.00E+00
8	19.13	2.33E+05	23.22	1.43E+04	25.98	2.18E+03	29.09	2.61E+02	33.62	1.19E+01	35.51	3.27E+00	ND	0.00E+00	ND	0.00E+00
9	18.76	3.00E+05	23.23	1.42E+04	25.63	2.76E+03	29.37	2.15E+02	33.11	1.68E+01	35.25	3.90E+00	ND	0.00E+00	ND	0.00E+00
10	19.01	2.53E+05	23.26	1.39E+04	25.85	2.38E+03	29.2	2.42E+02	33.01	1.80E+01	35.93	2.45E+00	ND	0.00E+00	38.33	3.06E-01
**mean**	19.02	2.54E+05	23.29	1.38E+04	25.85	2.40E+03	29.4	2.13E+02	33.22	1.60E+01	35.97	2.55E+00	38.14	3.07E-01	37.89	8.81E-02
**RSD%**	1.18%	14.64%	1.12%	14.77%	0.55%	9.76%	0.68%	13.51%	1.04%	23.89%	1.72%	35.23%	2.45%	120.37%	1.64%	222.72%
**No pos.**	10/10		10/10		10/10		10/10		10/10		10/10		5/10		2/10	

ND= undetermined

Supplementary Table 5. Determination of intra-assay variation.

	Copies/µL		
	**Sample1**	**Sample2**	**Sample3**
**Replicate 1**	2.46E+05	7.67E+01	8.36E+03
**Replicate 2**	1.94E+05	7.68E+01	6.80E+03
**Replicate 3**	2.05E+05	9.06E+01	7.35E+03
**mean**	2.15E+05	8.14E+01	7.50E+03
**RSD%**	12.75%	9.83%	10.54%

Supplementary Table 6. Determination of inter-assay variation.

	Copies/µL		
	**Sample 1**	**Sample 2**	**Sample 3**
**Run1**	5.71E+04	7.15E+05	2.26E+03
**Run 2**	7.78E+04	8.08E+05	2.86E+03
**Run 3**	6.51E+04	7.53E+05	2.80E+03
**Run 4**	7.14E+04	8.55E+05	1.96E+03
**mean**	6.79E+04	7.83E+05	2.47E+03
**RSD%**	13.04%	7.85%	17.57%
