# Supplementary material for: Porcine Epidemic Diarrhea Virus Shedding and Antibody Response in Swine Farms: A Longitudinal Study
Source: Front Microbiol. 2016 Dec 15;7:2009. doi: 10.3389/fmicb.2016.02009 (PMC5156881; doi:10.3389/fmicb.2016.02009)
Supplement: Supplementary file 2 [file Data_Sheet_2.pdf]

Supplementary Table 7. MIQE checklist (Bustin *et al*, 2009)

Item to check	Importance	checklist
**Experimental design**		
Definition of experimental groups	E	Materials & Methods
Number within each group	E	Materials & Methods
Assay carried out by the core or investigator's laboratory?	D	ND
Acknowledgment of authors' contributions	D	ND
**Sample**		
Description	E	Materials & Methods
Volume/mass of sample processed	D	Materials & Methods
Microdissection or macrodissection	E	NA
Processing procedure	E	Materials & Methods
If frozen, how and how quickly?	E	Materials & Methods
If fixed, with what and how quickly?	E	NA
Sample storage conditions and duration (especially for FFPEb samples)	E	Materials & Methods
**Nucleic acid extraction**		
Procedure and/or instrumentation	E	Materials & Methods
Name of kit and details of any modifications	E	Materials & Methods
Source of additional reagents used	D	NA
Details of DNase or RNase treatment	E	NA
Contamination assessment (DNA or RNA)	E	ND
Nucleic acid quantification	E	Materials & Methods
Instrument and method	E	Materials & Methods
Purity (A_{260}/A_{280})	D	ND
Yield	D	ND
RNA integrity: method instrument	E	ND
RIN/RQI or Cq of 3' and 5' transcripts	E	ND
Electrophoresis traces	D	NA
Inhibition testing (Cq dilutions, spike, or other)	E	Materials & Methods
**Reverse transcription**		
Complete reaction conditions	E	Materials & Methods
Amount of RNA and reaction volume	E	Materials & Methods
Priming oligonucleotide (if using GSP) and concentration	E	NA
Reverse transcriptase and concentration	E	NA
Temperature and time	E	Materials & Methods
Manufacturer of reagents and catalogue numbers	D	Materials & Methods
Cqs with and without reverse transcription	D	NA
Storage conditions of cDNA	D	NA
**qPCR target information**		
Gene symbol	E	Materials & Methods
Sequence accession number	E	Materials & Methods
Location of amplicon	D	Materials & Methods
Amplicon length	E	Materials & Methods
In silico specificity screen (BLAST, and so on)	E	Materials & Methods
Pseudogenes, retropseudogenes, or other homologs?	D	NA
Sequence alignment	D	ND
Secondary structure analysis of amplicon	D	ND
Location of each primer by exon or intron (if applicable)	E	NA

Item to check	Importance	checklist
**qPCR oligonucleotides**		
Primer sequences	E	Materials & Methods
RTPrimerDB identification number	D	NA
Probe sequences	D	Materials & Methods
Location and identity of any modifications	E	Materials & Methods
Manufacturer of oligonucleotides	D	ND
Purification method	D	ND
**qPCR protocol**		
Complete reaction conditions	E	Materials & Methods
Reaction volume and amount of cDNA	E	Materials & Methods
Primer, (probe), Mg^{2+}, and dNTP concentrations	E	Materials & Methods
Polymerase identity and concentration	E	Materials & Methods
Buffer/kit identity and manufacturer	E	Materials & Methods
Exact chemical composition of the buffer	D	Proprietary
Additives (SYBR Green I, DMSO, and so forth)	E	NA
Manufacturer of plates/tubes and catalog number	D	ND
Complete thermocycling parameters	E	Materials & Methods
Reaction setup (manual/robotic)	D	Materials & Methods
Manufacturer of qPCR instrument	E	Materials & Methods
**qPCR validation**		
Evidence of optimization (from gradients)	D	Materials & Methods
Specificity (gel, sequence, melt, or digest)	E	Materials & Methods
For SYBR Green I, Cq of the NTC	E	NA
Calibration curves with slope and y intercept	E	Table 3
PCR efficiency calculated from slope	E	Table 3
Confidence Intervals for PCR efficiency or Standard Error	D	ND
R^2 of calibration curve	E	Table 3
Linear dynamic range	E	Table 3
Cq variation at LOD	E	Table 3
Confidence Intervals throughout range	D	ND
Evidence for LOD	E	Table S4
If multiplex, efficiency and LOD of each assay	E	NA
**Data analysis**		
qPCR analysis program (source, version)	E	Materials & Methods
Method of Cq determination	E	Materials & Methods
Outlier identification and disposition	E	ND
Results for NTCs	E	Materials & Methods
Justification of number and choice of reference genes	E	NA
Description of normalization method	E	NA
Number and concordance of biological replicates	D	ND
Number and stage (reverse transcription or qPCR) of technical replicates	E	
Repeatability (intra-assay variation)	E	Table 3, Table S5
Reproducibility (inter-assay variation, CV)	D	Table 3, Table S6
Power analysis	D	ND
Statistical methods for results significance	E	Materials & Methods
Software (source, version)	E	Materials & Methods

What splice variants are targeted?	E	NA	Cq or raw data submission with RDML	D	ND

NA= Not Applicable, ND= Not Determine
