# Supplementary figures and images for: Porcine Epidemic Diarrhea Virus Shedding and Antibody Response in Swine Farms: A Longitudinal Study
Source: Front Microbiol. 2016 Dec 15;7:2009. doi: 10.3389/fmicb.2016.02009 (PMC5156881; doi:10.3389/fmicb.2016.02009)

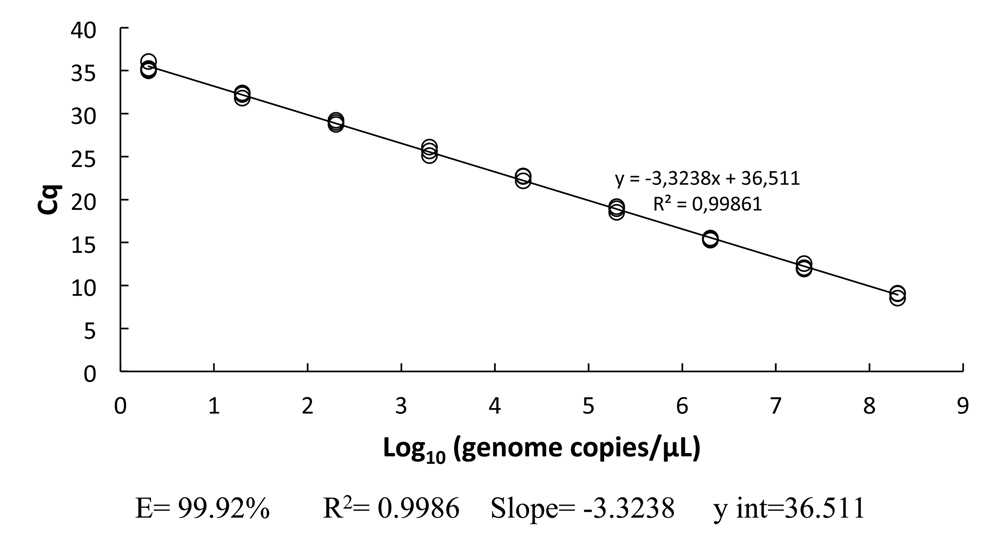

Supplement: Supplementary file 3 [file Image_1.JPEG]
